# Traumatic Brain Injury and Its Ophthalmologic Implications

**DOI:** 10.22336/rjo.2025.75

**Published:** 2025

**Authors:** Vlad Liviu Hârtie, Nicoleta Anton, Otilia Boișteanu, Maria Paula Comanescu, Emilia Pătrășcanu, Emilia Bologa, Daniela Șulea, Ciprian Danielescu, Mihaela Dana Turliuc

**Affiliations:** 1 “Gr. T. Popa” University of Medicine and Pharmacy, Iași, Romania; 2 “Prof. Dr. N. Oblu” Emergency Clinical Hospital, Iași, Romania; 3 “St. Spiridon” Emergency Clinical Hospital, Iași, Romania; 4 Regional Institute of Oncology, Iași, Romania

**Keywords:** traumatic brain injury, traumatic optic neuropathy, chronic traumatic encephalopathy, TBI = traumatic brain injury, TON = traumatic optic neuropathy, SD-OCTA = spectral domain optical coherence tomography, CTE = chronic traumatic encephalopathy, CSF = cerebrospinal fluid, RNFL = retinal nerve fiber layer, dLGN = dorsal lateral geniculate nucleus, IVMP = intravenous methylprednisolone

## Abstract

“Traumatic brain injury (TBI) frequently produces visual symptoms along the visual pathway, from the eye and optic nerve to cortical visual processing and oculomotor control. Manifestations include decreased visual acuity and visual fields, traumatic optic neuropathy (TON), oculomotor dysfunctions (saccades, vergence, tracking), photophobia/“visual snow”, and higher-level visuo-perceptual problems. Recognition of these symptoms by ophthalmologists and optometrists is essential, as many deficits are treatable (vision therapy, optical aids, medical/surgical management for TON, multidisciplinary rehabilitation), yet underdiagnosis is common. Significant gaps include standardized diagnostic protocols, objective biomarkers, and high-quality studies on rehabilitation strategies.

Recent advances in imaging methods allow noninvasive quantification of retinal thickness, retinal vessel density, blood flow, and oxygen saturation. SD-OCTA provides high-resolution three-dimensional (3D) images of blood flow and vessel density in the superficial and deep retinal plexuses. This novel, noninvasive imaging technique may offer insights into neural pathologies with vascular components, such as TBI.

## Introduction

Traumatic brain injury (TBI) represents a significant category of injuries resulting from direct impact to the head or from forces that cause sudden movement of the brain within the skull. These injuries can range from mild forms, such as a concussion, to severe, life-threatening damage that may lead to long-term neurological consequences. TBIs are an essential public health issue, being among the leading causes of disability and mortality, particularly in young individuals and in people involved in road accidents, falls, or sports activities. Understanding the mechanisms of injury, symptoms, and general principles of prevention significantly reduces the impact of these traumas on both individuals and society.

TBIs can be classified by severity using the Glasgow Coma Scale, which assesses eye, verbal, and motor responses to describe the patient’s level of consciousness on a scale from 3 to 15. A Glasgow score of 13–15 indicates a mild traumatic brain injury, a score of 9–12 suggests a moderate injury, and a score of 3–8 indicates a severe injury [**1**].

Following a high-velocity trauma, it is recommended to assess possible associated injuries in patients with traumatic brain injury (TBI), as the incidence of such injuries can reach up to 78%, and they may negatively influence the rehabilitation process and the patient’s long-term outcome [**2**].

Visual complications occurring after a traumatic brain injury, although not life-threatening, affect visual function and interfere with daily activities. The resulting injuries and functional deficits may vary depending on two major factors: whether the trauma was caused by blast or non-blast mechanisms, and whether the injury was penetrating or non-penetrating. Blast-related injuries are more than twice as likely to cause visual complications compared to non-blast injuries [**3**].

In penetrating injuries, the visual pathways and structures may be exposed to physical damage, such as lacerations caused by fractured bone fragments or foreign bodies. In non-penetrating injuries, also known as closed-head trauma, damage to the visual systems may occur due to displacement, stretching, or shearing forces [**4**]. Both mechanisms can lead to visual deficits; many regions of the cortex involved in visual processing are in areas vulnerable to traumatic brain injury, and nearly 70% of the brain’s sensory processing is related to vision [**5**].

Recent innovations in ophthalmic imaging have enabled the visualization of specific relationships between tissue structure and function in a variety of ophthalmologic and neurodegenerative diseases. Because the eye and the brain share critical embryological and physiological pathways, ocular imaging methods may provide a novel and valuable tool for improving the assessment of traumatic brain injury [**6**].

## Epidemiology

Often referred to as a silent epidemic, the long-lasting effects of traumatic brain injury (TBI)—including alterations in cognition, sensation, language, and emotions—are not always easily noticeable. In addition, public awareness regarding TBI appears to be limited, and long-term disability following such an injury often goes unrecognized [**7**].

It is estimated that over 10 million people suffer a traumatic brain injury (TBI) worldwide each year. In the United States, approximately 1.7 million people sustain a TBI annually; nearly 80% of these patients are treated in emergency departments, 16% are hospitalized, and 3% lose their lives [**8**]. Romania reports over 60,000 new cases of traumatic brain injury every year, corresponding to roughly 300 per 100,000 inhabitants. Patients with traumatic brain injuries often require prolonged medical and surgical treatment, including emergency care and hospitalization, which places a significant burden on the human and material resources of the Romanian healthcare system.

Traumatic brain injuries are associated with an increased risk of psychiatric disorders, including depression and anxiety, which may complicate recovery and rehabilitation.

## Objectives

This article reviews the current literature and data on ocular changes following traumatic brain injury (TBI). The main objective is to correlate the ocular alterations that arise following traumatic brain injury with the type and location of the trauma. Additionally, this article aims to examine the potential use of non-invasive ocular tests to diagnose or predict the prognosis of traumatic brain injury.

## Materials and methods

The electronic databases used in the search strategy included all relevant articles from PubMed, Google Scholar, and Web of Science, with all publication dates included up to November 2025. The inclusion criteria for journal articles were based on acute traumatic brain injuries, ocular disorders, diagnostic approaches, repetitive brain injuries, and blast-related trauma. Exclusion criteria included congenital brain injuries and pediatric patients. A total of 12 articles meeting the inclusion criteria were analyzed (**Table 1**).

**Table 1:** Articles that study ocular pathology in the context of Traumatic Brain Injury

Study Objective	Author, Year of Publication	Conclusions
**Quantitative pupillometry correlated with intracranial pressure (ICP) monitoring in TBI**	Martinez-Palacios K et al., 2024	Some parameters derived from quantitative pupillometry (NPi, CV, and MCV) appear to play a role in predicting and staging intracranial hypertension
**Investigation of nystagmus in the context of traumatic brain injury**	de Clercq H et al., 2017	All patients with acute traumatic brain injury should be examined ophthalmologically and vestibularly
**Retinal manifestations in acute traumatic brain injury**	Laws E et al., 2025	Reports an association between longitudinal changes in retinal nerve fiber and TBI severity
**Disconjugate oculomotor changes associated with structural traumatic brain injury**	Samadani U et al., 2015	The applied protocol correlates the presence of TBI in study patients with a high risk of oculomotor changes
**Complement system activation promotes ocular pathology after traumatic brain injury**	Borucki DM et al., 2024	Data indicate that complement activation plays a vital role in the development of visual deficits after TBI
**Traumatic brain injury and dry eye syndrome among U.S. veterans**	Lee CJ et al., 2017	Dry eye syndrome and chronic pain syndromes occur more frequently in patients diagnosed with TBI, suggesting common pathophysiological aspects
**Microglial activation and Caspase 3 in the retina after TBI**	Kovacs-Oller T et al., 2023	Increased levels of microglial cells and Caspase 3 positive cells in the retina after TBI suggest increased inflammation and cell death
**Impact of minor TBI on reading comprehension and eye movements**	Ratiu I et al., 2022	The group of TBI patients showed a lower frequency of blinking, which may mean they required a higher level of cognitive resources
**Traumatic optic neuropathy**	Blanch RJ, 2024	Intravenous corticosteroid use has an adverse effect, and optic canal decompression shows no beneficial effect in traumatic optic neuropathy
**Eye movements during reading after TBI**	Reddy AVC et al., 2019	Significant decrease in eye movements during reading for TBI patients compared to the control group
**Visual function after polytrauma**	Goodrich GL et al., 2007	The rate of visual deficits after blast injuries was 52%, compared to 20% for other types of injuries
**Chronic traumatic encephalopathy in athletes**	McKee AC et al., 2009	Chronic traumatic encephalopathy is a pathology caused by tau proteins, with a clearly acquired etiology

### Structural changes in the context of traumatic brain injury

TBI includes cerebral contusions caused by coup and contrecoup injuries, resulting from the impact of the brain against the skull. More severe injuries may be caused by external forces, which can lead to subdural or epidural hemorrhage, subarachnoid hemorrhage, and shearing of nerve fibers. Repetitive trauma can cause an acute-phase concussion. More importantly, it can lead to chronic traumatic encephalopathy (CTE), with tau protein deposition, which in turn may result in neurodegeneration [**9**].

The mechanisms of brain injury following blast trauma range from the direct effects of shock waves on the cerebrospinal fluid (CSF) and pressure-related effects to the secondary impacts caused by debris that may even result in penetrating injuries [**10**]. The shock wave advancing within the confined space of the skull and meninges can cause cerebral edema, cerebral vasospasm, and diffuse axonal injury (DAI) [**11**]. In mild TBI, patients may experience neuropsychiatric symptoms, long-term cognitive disabilities, and other forms of post-traumatic stress disorder [**12**].

The use of noninvasive imaging to monitor TBI and explore biomarkers that may aid diagnosis is a current area of interest.

### Pupillary reactivity

Pupillary examination is an essential component of the neurological assessment. This includes evaluating the size and equality of the pupils, their shape, and their reaction to light. It is necessary to know whether the patient has undergone any ophthalmologic procedures, as these may influence the examination. For example, pupillary constriction is reduced after cataract surgery.

Light reactivity is assessed in both the ipsilateral and contralateral pupils and is documented as round and reactive to light, unilaterally or bilaterally nonreactive, fixed, and/or dilated. The speed of the pupillary response is described as brisk or sluggish.

In patients with TBI, the pupillary examination may initially be normal, but can change as intracranial pathology evolves, sometimes hours after the primary injury. Serial pupillary examinations are essential for detecting subtle changes in the patient’s neurological status.

Pupillary assessment is particularly useful because it is one of the few noninvasive neurological evaluations that can also be performed in unconscious, sedated, or paralyzed patients [**13**].

### Ocular changes in the context of traumatic brain injury

A retrospective study by Weichel and colleagues aimed to determine the impact of TBI on veterans with combat-related ocular trauma. The authors found that closed-globe injuries were more likely to be associated with concomitant brain injuries compared with open-globe injuries [**14**]. Patients report a wide range of visual symptoms following blast-induced TBI, including decreased visual acuity, visual field defects, difficulties coordinating eye movements, and higher-level deficits involving visual perception and visuospatial function [**15**]. Retinal injuries ranged from intraretinal hemorrhages to retinal detachment and choroidal rupture [**5**]. The reported rate of post-chiasmal visual field defects detected through manual techniques in TBI patients has ranged from 3.2% to 39% [**16**]. Inpatients reported more visual field defects than outpatients, and these visual field defects were more frequent in blast-related trauma than in non-blast injuries [**5**].

One significant visual field defect observed is homonymous hemianopia. In their study of 61 military personnel exposed to blast trauma, Lemke and colleagues found that 15% of participants exhibited hemianopic or quadrantanopic visual field defects, and 36% had abnormal global visual field indices [**17**]. Ocular complications arising from repeated head trauma, as observed in contact sports such as boxing and football, can range from asymptomatic lesions to severe vision-threatening complications such as retinal detachment. Commonly affected structures include the iris, lens, anterior chamber angle, retina, and optic nerve [**18**].

In their study of 25 active, asymptomatic boxers, Wedrich and colleagues found that 76% presented pathological structural ocular changes attributed to contusive trauma. Twenty-eight percent of boxers with anterior segment injuries also had posterior segment injuries. Peripheral retinal scars were the most common abnormality observed (60%). Other posterior segment abnormalities included posterior vitreous detachment (12%) and retinal tears or holes (24%). Significant correlations were found between the total number of matches, the total number of losses, and the presence of retinal tears [**19**].

### Chronic traumatic encephalopathy (CTE)

Studies were done to better understand the long-term effects of a single head injury compared with repetitive brain injuries, how repeated TBIs can lead to CTE, and how frequently these changes occur in adults [**20**].

In a postmortem study, McKee and colleagues examined brains that had sustained repeated mild traumatic brain injuries. They divided the pathology into four stages. In Stage 1, the macroscopic appearance is normal, while microscopically, there is perivascular tau protein as well as neurofibrillary and astrocytic tangles.

Stages 2 and 3 show mild to moderate ventricular enlargement on gross examination. Microscopically, the presence of tau protein and neurofibrillary tangles increases with severity. These changes are predominant in the frontal and temporal lobes and in the hippocampus.

Cerebral atrophy begins in Stage 3, and in Stage 4, atrophy becomes more pronounced, with ventricular enlargement associated with reduced brain weight. Extensive tau protein deposition and neurofibrillary tangles are observed [**21**].

### Complement system activation in TBI – ophthalmologic considerations

Borucki DM et al. demonstrated in 2024 the correlation between complement system activation following acute traumatic brain injury and the subsequent ocular changes. Acute and chronic neuroinflammatory changes in the dorsal lateral geniculate nucleus (dLGN) and the retina were investigated after a unilateral, moderate-to-severe controlled cortical impact in mice. Neuroinflammatory and histopathological results were interpreted in the context of behavioral and visual function data. To examine the role of complement, certain groups were treated after TBI with the complement inhibitor CR2-Crry.

TBI induces complement activation within the dLGN and stimulates microglial activation and synaptic internalization. Complement inhibition after TBI, in a clinically relevant model, reduces complement activation, maintains a microglial phenotype closer to a surveillance state, and preserves synaptic density in the dLGN.

Overall, the data indicate that the complement system plays a key role in the development of visual deficits after TBI through complement-dependent microglial phagocytosis of synapses in the dLGN [**22**].

### Traumatic optic neuropathy

Traumatic optic neuropathy is classically described in up to 8% of patients with traumatic brain injury (TBI), but subclinical or undiagnosed optic nerve damage is far more common. When more sensitive tests are used, at least half of patients with moderate-to-severe TBI show visual field defects or optic atrophy on optical coherence tomography.

Acute compression of the optic nerve and ischemia in the context of orbital compartment syndrome require urgent surgical and medical intervention to reduce intraocular pressure and minimize the risk of permanent optic nerve dysfunction. Other manifestations of traumatic optic neuropathy are treated with more variability across international medical practice.

In 2024, Blanch RJ et al. demonstrated through a systematic review that there is no evidence supporting the benefit of any medical treatment for traumatic optic neuropathy. Still, there is strong evidence of harm associated with intravenous methylprednisolone (IVMP). Likewise, there is no evidence supporting the benefit of optic canal decompression in traumatic optic neuropathy. Orbital compartment syndrome is a distinct entity that requires both medical and surgical intervention to prevent vision loss [**23**].

Moreover, in the context of traumatic brain injury, visual snow syndrome can occur. This is a neurological disorder in which a person continuously sees tiny flickering dots – similar to television static – across their entire field of vision. It is always present, even with eyes closed, and is not caused by an eye disease.

### The retina and acute traumatic brain injury

The retina is the tissue that contains the ganglion cells responsible for receiving and transmitting visual stimuli to the brain. In TBI, inflammation caused by microglial proliferation following blast injuries can lead to visual deficits among military personnel [**24**]. Rodent studies, involving both repetitive brain injuries and blast-induced trauma, have shown a reduction in optic nerve diameter and thinning of the retinal nerve fiber layer (RNFL) after TBI [**25**]. Research conducted on blast-induced animal models (mice) has demonstrated increased activation of inflammatory markers and glial fibrillary acidic protein, as well as loss of retinal ganglion cells (RGCs). An *in vitro* study using adipose-derived stem cells pre-stimulated with inflammatory cytokines showed that these cells reduced visual dysfunction after blast injuries by decreasing inflammatory cytokine expression [**24**].

In a study that monitored 16 Olympic boxers over 18 months, researchers observed macular thinning and RNFL thinning on OCT examination, compared with healthy, sedentary control subjects [**26**]. In patients with acquired injuries to the post-geniculate visual pathways, a reduction in circumpapillary RNFL thickness corresponding to hemianopic visual field loss was detected using spectral-domain optical coherence tomography (SD-OCT). Furthermore, this change was more pronounced at 24 months than at the initial visit. This progression suggests that retrograde trans-synaptic degeneration (RTSD) is at least partially responsible for RNFL thinning after TBI [**27**]. Evidence of retinal ganglion cell (RGC) damage is not always detectable initially, especially when the injury is minor [**28**]. Trans-synaptic neuronal degeneration can evolve, increasing permanent disability long after the initial central nervous system injury. Although the limits of this cascade are not fully known, recent findings suggest that when trans-synaptic degeneration occurs in a retrograde direction, it does not extend beyond a single synaptic neuron [**29**].

When performing retinal imaging to detect injury after TBI, it is essential to determine which retinal region will provide the most consistent and accurate results. Layer segmentation has allowed a more detailed study of functional zones. The macula contains the highest density of retinal ganglion cells (RGCs) and may therefore be the optimal location for early detection of RGC injury. Additionally, optic disc edema can obscure axonal loss at the optic nerve head, making the macula an even more suitable candidate for RGC analysis [**30**].

Retinal oximetry is a noninvasive ocular imaging technique that enables the assessment of oxygen content and extraction in the major retinal blood vessels. Retinal oximetry may help elucidate how TBI affects oxygen delivery and utilization in the retina. Specifically, this technique provides information on retinal oxygen use and, by extension, retinal metabolism, by measuring the relative amounts of oxyhemoglobin (HbO_2_) and deoxyhemoglobin (Hb) in retinal vessels [**31**]. After TBI, changes in microcirculation are multifactorial. For example, affected and edematous tissue has been reported to compress extrinsic microvasculature, triggering contraction of smooth muscle in resistance arterioles and inducing intravascular thrombosis [**32**].

Recent advances in imaging methods—such as SD-OCT, SD-OCT angiography (SD-OCTA), Heidelberg retinal flowmetry (HRF), and retinal oximetry—allow noninvasive imaging and quantification of retinal thickness, retinal vessel density, blood flow, and oxygen saturation. SD-OCTA provides high-resolution three-dimensional (3D) images of blood flow and vessel density in the superficial and deep retinal plexuses. This novel, noninvasive imaging technique may offer valuable insight into neural pathologies with vascular components, such as TBI [**6**].

## Conclusions

TBI produces a broad spectrum of ophthalmologic and neuro-ophthalmologic pathophysiological changes through diverse mechanisms. Many visual sequelae are underrecognized and treatable; ophthalmology specialists play a central role in diagnosis, monitoring, and multidisciplinary rehabilitation. Priority research areas include the development of standardized and objective assessment tools, more robust clinical studies on rehabilitation therapies, and the identification of better biomarkers to guide prognosis and interventions in TBI.
